# A Candidate MEST Splice-Site Variant in a Patient with Silver–Russell Syndrome-like Phenotype: First Report and Literature Review

**DOI:** 10.3390/genes17090992

**Published:** 2026-08-24

**Authors:** Xiaocha Xu, Rongrong Pan, Shuai Chen, Fan Yu, Haixia Miao, Kexin Fang, Dingwen Wu, Yi Zhang, Jing Li, Xin Yang

**Affiliations:** 1 Department of Genetics and Metabolism, Children’s Hospital, Zhejiang University School of Medicine, National Clinical Research Center for Children and Adolescents’ Health and Disease, Hangzhou 310051, China; 2 Pediatric Healthcare Department, Jiaxing Maternity and Child Health Care Hospital, Jiaxing 314000, China; 3 Zhejiang Key Laboratory of Neonatal Diseases, Hangzhou 310052, China; 4 Binjiang Institute of Zhejiang University, Hangzhou 310053, China

**Keywords:** *MEST* gene, Silver–Russell syndrome, splice-site variant, genomic imprinting, growth restriction

## Abstract

Silver–Russell syndrome (SRS) is most commonly caused by epigenetic alterations at 11p15.5 or maternal uniparental disomy of chromosome 7 [upd(7)mat], though other molecular mechanisms remain unclear. While microdeletions encompassing *MEST* have been associated with SRS-like phenotypes, no pathogenic intragenic *MEST* variants have been reported to date. We describe a 6-month-old male infant with clinical features suggestive of a SRS-like *phenotype*, including intrauterine and postnatal growth restriction, triangular facies, prominent forehead, and small extremities. Methylation-specific multiplex ligation-dependent probe amplification (MS-MLPA) revealed neither methylation abnormalities at 11p15.5, 7p13, or 7q32 nor copy number variations (CNVs) in these regions. Trio whole-exome sequencing (trio-WES) identified a paternally inherited splice-site variant (c.890 + 1G > A) in *MEST*. Given the paternal-specific expression of *MEST*, this variant resides on the functionally active allele. Based on in silico predictions and clinical correlation, this case identifies *MEST* as a plausible candidate gene for SRS and provides a rationale for further functional studies. Phenotypic variation exists across molecular subtypes, yet definitive genotype–phenotype correlations await larger, systematically ascertained cohorts.

## 1. Introduction

*MEST* (also known as *PEG1*), located at 7q32.2, is a paternally expressed imprinted gene encoding a member of the α/β-hydrolase superfamily. Its specific enzymatic substrates remain poorly characterized. *MEST* expression is regulated by a differentially methylated region (DMR): the maternal allele is silenced by DMR hypermethylation, while the paternal allele is actively transcribed. *MEST* is highly expressed in placental trophoblasts and endothelial cells, implicating it in placental development and fetal growth regulation [1].

*Mest* knockout mice exhibit reduced placental weight, late-onset fetal growth restriction, and postnatal growth restriction; these features are observed upon paternal transmission of the mutant allele, consistent with paternal-specific expression [2]. These murine data support loss of *MEST* function as a plausible disease mechanism for growth disorders. *MEST* is also involved in metabolic regulation; its adipose tissue expression is elevated in obesity and further upregulated in ob/ob and db/db mice [3]. Furthermore, this gene plays an essential role in digit regeneration: conditional knockout mice show impaired bone regrowth following digit-tip amputation, potentially via modulation of neutrophil responses [4].

Silver–Russell syndrome (SRS, OMIM 180860) is a rare imprinting disorder with an estimated incidence of 1 in 30,000 to 1 in 100,000 live births [5]. Core features include intrauterine and postnatal failure to thrive, relative macrocephaly, triangular facies, prominent forehead, body asymmetry, and feeding difficulties [5,6]. The genetic aetiology is heterogeneous. Major causes include 11p15.5 imprinting abnormalities (30–60%), upd(7)mat (5–10%), and pathogenic variants in *IGF2*, *PLAG1*, and *HMGA2* [7,8]. Other rare molecular subtypes include upd(14)mat, upd(16)mat, and upd(20)mat [7]. Despite extensive screening, a substantial proportion of cases remain molecularly unexplained. Altered *MEST* expression at 7q32 has been associated with the typical SRS manifestations in patients with upd(7)mat [9], indicating a role for *MEST* in SRS pathogenesis. *MEST* locus microdeletions [10,11,12] and aberrant *MEST* DMR methylation [13] have been linked to SRS-like phenotypes, suggesting that haploinsufficiency of the paternally expressed *MEST* allele may contribute to the pathogenesis of growth disorders. However, previous coding sequence analyses failed to identify pathogenic variants [14,15], and whether intragenic *MEST* variants directly cause SRS in humans remains unknown.

Here, we report the first patient with an SRS-like phenotype carrying an intragenic *MEST* splice-site variant (*NM_002402.4*: c.890 + 1G > A). Located at the canonical splice donor site, this variant is predicted to impair normal splicing and possibly result in functional loss, pending experimental confirmation. Our case supports a possible contribution of this intragenic *MEST* variant to SRS-like pathogenesis.

## 2. Methods

*SNV analysis.* Trio whole-exome sequencing (trio-WES) was performed on the proband and parents using the KAPA HyperExome V2 Probes kit (Roche Molecular Systems, Inc., Pleasanton, CA, USA), with paired-end 150-bp sequencing on the MGI DNBSEQ-T7 platform (MGI Tech., Beijing, China). Reads were aligned to GRCh37/hg19 using BWA v2.2.1, and variants were called (GATK v4.0) and annotated (VEP v113, ANNOVAR 2020-06-08). Mean coverage was ≥100×, with >95% of target bases at ≥20×. Variants were filtered against gnomAD v2.1.1, dbSNP, and an in-house database; common variants (MAF > 1%) and ClinVar benign/likely benign entries were excluded. A stringent MAF threshold of <0.01% was applied to known SRS-associated and SRS-phenocopy genes (*IGF2*, *H19*, *CDKN1C*, *PLAG1*, *HMGA2*, *MEST*, *GRB10*, and *IGF1R*). Pathogenicity was predicted using SIFT, PolyPhen-2, MutationTaster, REVEL, and CADD for missense variants, and SpliceAI, MaxEntScan, and dbscSNV for splice-site variants. All variants were classified per ACMG/AMP 2015 guidelines and prioritized by phenotypic concordance, inheritance pattern, in silico scores, and sequencing quality.

*Copy number variations (CNVs) and UPD analysis.* CNVs were called from WES data using ExomeDepth and cn.MOPS; only concordant calls passing quality thresholds (mean coverage ≥ 100×, Pearson correlation > 0.97, Bayes factor ≥ 10, posterior probability ≥ 0.95) were retained. CNVs were filtered against population databases (frequency < 0.1% for duplications, <0.05% for deletions), except those annotated as pathogenic/likely pathogenic in ClinVar. Pathogenicity was classified per ACMG/ClinGen criteria. UPD was assessed by Mendelian inheritance analysis of informative SNPs from trio-WES data across imprinted regions on chromosomes 7 and 11 (11p15.5, 7p13, 7q32), compared with parental genotypes. The number and distribution of informative markers were dependent on WES capture design and coverage within these regions; this region-specific analysis did not include chromosome-wide SNP evaluation. No evidence of UPD was detected within the limitations of the methods used; however, maternal UPD7, particularly heterodisomy, cannot be fully excluded. Normal MS-MLPA findings do not independently exclude all forms of chromosome 7 UPD.

*Methylation-specific multiplex ligation-dependent probe amplification (MS-MLPA).* Copy number and methylation status of imprinted regions were assessed using the SALSA MLPA Reagent Kit (MRC-Holland). Probe mixes were hybridized to genomic DNA; undigested and HhaI-digested aliquots were processed for copy number and methylation analysis, respectively. Products were separated by capillary electrophoresis and analyzed using Coffalyser.Net. Normal MS-MLPA results do not exclude all forms of chromosome 7 UPD.

*Variant validation.* Candidate variants and familial segregation were confirmed by Sanger sequencing.

*Growth assessment.* Growth parameters were expressed as SDS using the INTERGROWTH-21st Newborn Size Standards for gestational age [16].

## 3. Case Presentation

The proband is a male infant, the first child of healthy, non-consanguineous parents. The mother had a history of gestational diabetes mellitus and polycystic ovary syndrome. Prenatal ultrasonography revealed a velamentous umbilical cord insertion. The infant was delivered via caesarean section at 37 weeks of gestation due to this placental abnormality. Birth measurements were markedly reduced: birth weight 1.95 kg (SDS −2.7) and birth length 42 cm (SDS −3.5), consistent with severe small-for-gestational-age status. Head circumference at birth was not recorded.

The infant exhibited persistent growth failure from birth. At the initial paediatric evaluation at 6 months of age, his weight was 4.7 kg (SDS −4.0), length 57 cm (SDS −4.5), and head circumference 41 cm (SDS −1.5). He had significant feeding difficulties from early infancy; despite frequent breastfeeding every 2–3 h, intake was insufficient, with each session lasting 20–30 min, and occasional milk regurgitation without haematemesis. These feeding challenges necessitated repeated nutritional support, including prolonged administration of lysine and zinc sulfate oral solution and intermittent iron supplementation. Catch-up growth had not been achieved despite these interventions.

Characteristic dysmorphic features included frontal bossing, fine eyebrows, a long philtrum, a thin upper lip, a wide mouth, and a short neck. The fingers were short, with a single palmar crease on the left hand. The feet were small, with mild dorsal oedema. The body was symmetrical, with no limb length discrepancy. Hip examination was unremarkable (Ortolani negative), and muscle tone was normal. No hepatosplenomegaly or abdominal masses were present. Motor development was at the lower limit of normal; at 6 months of age he could sit briefly with support.

Newborn hearing screening was abnormal, requiring audiological follow-up. Auditory brainstem response testing at 7 months demonstrated a wave V threshold of 30 dB nHL in the left ear and 25 dB nHL in the right ear, indicating mild asymmetric hearing loss. By 10 months, thresholds improved to 20 dB nHL (left) and 15 dB nHL (right), corroborated by parental observations. Laboratory investigations revealed iron-deficiency anaemia (microcytic, hypochromic indices, low ferritin, reduced transferrin saturation), zinc deficiency, and mildly elevated β-hydroxybutyrate with normal glycaemic control (HbA1c 5.4%), consistent with physiological ketosis secondary to inadequate caloric intake. Electrolytes and thyroid function were normal; TORCH screening was negative. Abdominal ultrasonography and cranial MRI showed no structural abnormalities. Atopic dermatitis developed at 18 months and allergic rhinitis at 25 months. At 31 months, growth failure persisted despite prolonged nutritional support, iron supplementation, and dermatological care.

Clinical evaluation revealed features overlapping with SRS: severe intrauterine growth restriction (birth weight SDS −2.7, length SDS −3.5), persistent postnatal growth failure (6 months: weight SDS −4.0, length SDS −4.5), prominent forehead, and feeding difficulties requiring nutritional supplementation. Birth head circumference was unavailable, precluding formal assessment of relative macrocephaly and reliable application of the Netchine–Harbison clinical scoring system (NH-CSS) [6,17]. Body asymmetry was not observed. These findings prompted molecular testing.

**Figure 1:**
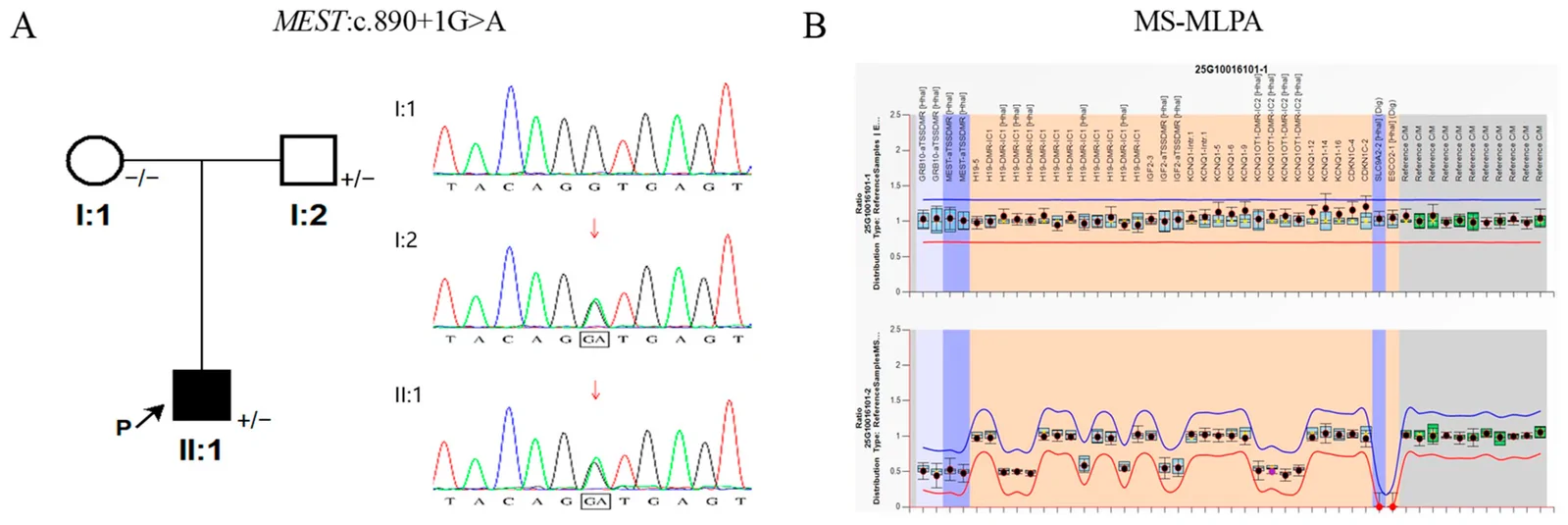
Genetic and epigenetic analysis. (**A**) Pedigree and *MEST* variant. (**B**) MS-MLPA of 11p15.5, 7p13, and 7q32 imprinted regions. No detectable copy number variations or methylation abnormalities were identified. Blue and red lines denote upper and lower copy-number ratio thresholds. Blue, orange and gray shading indicate the target-gene, target-probe and normalization reference-probe regions. Black dots show sample-to-reference probe ratios with vertical bars for 95% confidence intervals. Light-blue boxes mark normal-range probes; yellow asterisks denote mean values of reference samples; green boxes flag out-of-threshold probes. Green boxes in reference-probe regions reflect experimental variability rather than CNVs. Ratios above or below thresholds indicate duplication or deletion. Abnormal reference-probe markers serve only for quality-control assessment. The ellipsis on the y-axis originates from software text truncation.

Trio-WES detected a paternally inherited splice-site variant in *MEST* (*NM_002402.4*: c.890 + 1G > A; chr7:g.130143838G > A [GRCh37] or chr7:g.130503997G > A [GRCh38]; read depth: 142×; allelic balance: 0.50) in the proband, with familial segregation confirmed by Sanger sequencing. Per ACMG/AMP criteria [18], this variant was classified as a variant of uncertain significance (VUS). No clinically significant CNVs were identified in the proband or parents. No evidence of uniparental disomy or extended regions of homozygosity was detected within the 11p15.5, 7p13, and 7q32 imprinted regions, although maternal UPD7 cannot be fully excluded. MS-MLPA showed no detectable methylation abnormalities or copy number variations in these regions (Figure 1). Incidental findings included homozygous *GJB2* c.109G > A (p.V37I) and compound heterozygous *SLC12A3* variants (c.781C > T and c.965-1_976delinsACCGAAAATTTT), unrelated to the SRS phenotype.

## 4. Discussion

We systematically compared clinical presentations across major SRS molecular etiologies—11p15 LOM, upd(7)mat, segmental upd(7q)mat, and *MEST*-related alterations (microdeletions and intragenic variants) (Table 1)—revealing descriptive phenotypic differences. No formal severity gradient can be inferred given heterogeneous sample sizes, ascertainment methods, and molecular mechanisms.

Patients with 11p15 LOM exhibited the most severe and classic clinical presentation, with the highest frequencies of core features (IUGR, near 100%; postnatal growth restriction, approximately 84%; relative macrocephaly, approximately 99%; body asymmetry, approximately 77%; and clinodactyly, approximately 81%) [7]. Mechanistically, loss of methylation at ICR1 (H19/IGF2 IG-DMR) on the normally methylated paternal allele reduces *IGF2* expression and activates biallelic *H19* expression, thereby disrupting *IGF2*-mediated growth signaling [6,20,21].

Upd(7)mat-associated SRS shows intermediate severity, with high frequencies of core features (IUGR, 72.7%; postnatal growth restriction, 80.9%; feeding difficulties, 87.2%; and relative macrocephaly, 85.2%), but body asymmetry is markedly less frequent (29%) [7]. This indicates a substantial impact on growth and feeding, while structural anomalies are less prevalent. Mechanistically, this may reflect the combined silencing of paternally expressed genes (including loss of *MEST* expression at 7q32 [9]) and biallelic expression of maternally expressed genes across chromosome 7, representing a multilocus imprinting disturbance. Because upd(7)mat affects the entire chromosome rather than a discrete regulatory region, the molecular effect may be dispersed across multiple loci, potentially contributing to a milder overall phenotype than 11p15 LOM [9,22,23].

Segmental upd(7q)mat is associated with a narrower phenotypic spectrum. Of four reported patients, only two exhibited IUGR, whereas all four presented with postnatal growth restriction, feeding difficulties, and relative macrocephaly; none manifested body asymmetry [19]. This suggests that the 7q region, despite harboring key imprinted genes, may be insufficient alone to account for the full spectrum of SRS features. Upd(7q)mat recapitulates *MEST* loss through paternal allele absence, supporting *MEST* as a key candidate [9,19].

Further evidence comes from patients with *MEST* deletions. Three reported patients with microdeletions spanning the *MEST* locus (3.7 Mb [10], 2.8 Mb [11], and 79 kb [12]) all exhibited SRS-like phenotypes centered on growth and developmental impairment. Height SD ranged from −3.13 to −1.58, with feeding difficulties and relative macrocephaly as accompanying features, whereas typical facial features and body asymmetry were variable or absent. Notably, the 79-kb microdeletion reported by Vincent et al. encompassed only *CEP41*, *MEST*, *MESTIT1*, *MIR335*, and *COPG2*, with *MEST* as the most likely pathogenic candidate [12]. Phenotypic analyses of these deletion carriers support a central role for *MEST* in growth regulation, whereas its contribution to craniofacial features and body asymmetry appears minor or requires additional genetic factors. Whether intragenic *MEST* variants—rather than large deletions or chromosomal abnormalities—can directly cause SRS remained untested, a question this study addresses.

We identified an intragenic *MEST* splice-site variant (*NM_002402.4*: c.890 + 1G > A) in a patient with an SRS-like phenotype, expanding the molecular spectrum beyond previously reported large microdeletions at this locus. The canonical isoform *NM_002402.4* (isoform a) is the longest transcript, paternally expressed, and highly expressed in placenta and fetal tissues [1,24]; whether this pattern is conserved in all clinically relevant tissues throughout development remains incompletely characterized. According to ACMG/AMP 2015 guidelines, c.890 + 1G > A was classified as a VUS, reflecting the current evidence gap for *MEST*-specific disease causality. Only PM2 (absent/minor frequency in population databases) was reliably applicable. PP3 (splicing prediction) and PP4 (phenotypic matching) were applied cautiously due to the lack of functional validation and the heterogeneity inherent to SRS. PVS1 (null variant pathogenicity) was not applicable, as definitive human disease causality for *MEST* loss-of-function has not been formally established. All previously reported pathogenic *MEST*-related alterations were large paternal microdeletions spanning multiple dosage-sensitive genes and regulatory elements, rather than discrete intragenic splice variants. This variant is predicted to disrupt the canonical GT–AG splice motif at the invariant +1 position of the exon 11/intron 11 boundary. The disrupted donor site is evolutionarily conserved, and multiple in silico tools consistently support aberrant splicing. Predicted consequences include exon 11 skipping, cryptic splice-site activation, or intron 11 retention, any of which would disrupt the reading frame or introduce a premature termination codon, potentially triggering nonsense-mediated mRNA decay (NMD) and leading to reduced or absent MEST protein [25,26]. The highly conserved splice-site disruption, concordant in silico predictions, and documented role of *MEST* in fetal and placental growth provide strong biological plausibility for this variant as a causal candidate.

The variant was inherited from the phenotypically unaffected father. As *MEST* is paternally expressed, the variant in the proband resides on the active allele. The father’s normal phenotype may reflect carriage on his silent maternal allele; alternatively, if the variant resides on his active paternal allele, his unaffected status would suggest incomplete penetrance or variable expressivity. Neither scenario can be distinguished without grandparental testing, which was not feasible. Several caveats apply. The splice-disruption mechanism is based solely on in silico predictions and remains hypothetical pending functional validation—ideally encompassing RT-PCR, transcript sequencing, quantitative expression analysis, and allele-specific studies. If patient-derived RNA is unavailable, heterologous minigene splicing assays could provide supportive evidence. All pathogenic inferences remain preliminary and require definitive functional validation and replication in independent cohorts. Additional limitations include lack of validation in larger cohorts, unknown penetrance and phenotypic spectrum, and incomplete longitudinal data attributable to the proband’s young age and non-systematic clinical follow-up. Whether exon 11 is included in all major *MEST* isoforms, and whether its skipping affects all clinically relevant transcripts, remains undetermined. Normal *MEST* DMR methylation by MS-MLPA confirms epigenetic integrity but does not demonstrate transcriptional activity of the variant allele, nor that the affected transcript is the clinically relevant imprinted transcript. Allele-specific expression analysis was not performed, precluding confirmation that the variant-bearing allele is actively transcribed in growth-relevant tissues. Elucidation of *MEST* regulatory networks and their role in SRS pathogenesis therefore awaits further investigation.

Comparison of this case with three reported *MEST* deletion patients reveals substantial phenotypic overlap, with all four sharing growth impairment as a core feature; the three deletion patients also exhibited relative macrocephaly, whereas this could not be formally assessed in the present patient due to unavailable birth head circumference. Patients with large deletions presented with mild facial dysmorphism and multisystem congenital anomalies, whereas those with the 79-kb microdeletion or *MEST* splice variant showed predominantly growth-restricted phenotypes; however, this distribution is purely descriptive and cannot establish that deletion size determines clinical severity. The small number of cases, variable ascertainment, and potential contributions of other genes within the deleted regions (*CEP41*, *COPG2*, *MIR335*, and regulatory elements) preclude any firm genotype–phenotype correlation. None of the four *MEST*-related patients presented with body asymmetry—a hallmark of 11p15 LOM SRS present in approximately 77% of that subgroup—which is consistent with the hypothesis that *MEST* dysfunction preferentially impairs growth-regulatory pathways while sparing mechanisms governing body symmetry [6,20,27]. Neurodevelopmental trajectories were heterogeneous across reported *MEST* deletion carriers; longitudinal assessment for the current proband is pending. Collectively, these observations suggest that MEST primarily functions in the placental-fetal growth axis, whereas its independent effects on craniofacial morphology and neurodevelopment may be secondary and modulated by additional genetic modifiers.

While velamentous cord insertion and nutritional factors may have contributed to fetal growth restriction, the persistence of postnatal growth failure despite nutritional intervention, together with characteristic dysmorphic features, suggests an underlying genetic contribution. The paternally inherited *MEST* splice-site variant—in a gene with established roles in placental and fetal growth [1,2]—provides a plausible unifying explanation. Placental histopathological data were unavailable, precluding complete assessment of placental factors.

The patient also carried a homozygous *GJB2* c.109G > A (p.V37I) variant and biallelic pathogenic *SLC12A3* variants. Both are incidental findings unrelated to the SRS phenotype. The *GJB2* variant is common in East Asian populations and associated with mild sensorineural hearing loss [28]; however, the proband’s transient hearing screening abnormality resolved spontaneously by 10 months of age (ABR thresholds improved from 30/25 dB nHL to 20/15 dB nHL without intervention), consistent with transient conductive or developmental immaturity rather than permanent sensorineural loss. Its self-resolving course and lack of progressive impairment render it an isolated incidental finding, unrelated to either the *GJB2* variant or the *MEST*-associated phenotype. Biallelic *SLC12A3* variants cause Gitelman syndrome, typically presenting from late childhood with hypokalaemia, hypomagnesaemia, and metabolic alkalosis [29,30]; although currently asymptomatic, lifelong electrolyte surveillance is warranted.

## 5. Conclusions

This study extends prior evidence for *MEST*-related SRS—previously restricted to large deletions and chromosomal abnormalities—to intragenic loss-of-function variants (c.890 + 1G > A; pending functional validation). Two principal conclusions emerge. First, *MEST* is implicated as a plausible candidate gene for SRS-like growth disorders, underscoring the utility of WES in identifying rare single-gene defects in imprinted genes beyond conventional first-tier (epi)genetic testing. Second, while descriptive comparisons across molecular subtypes reveal phenotypic heterogeneity, the limited and heterogeneous nature of available cases precludes any firm inference of genotype–phenotype correlations or severity gradients.

## Figures and Tables

**Table 1:** Comparison of major clinical features between the *MEST* splicing variant patient, three patients with 7q32.2 microdeletions, and other SRS genotypes.

	Total SRS [7] (*n*)	11p15 LOM [7] (*n*)	upd(7)mat [7] (*n*)	Segmental upd(7q)mat [19] (*n* = 4)	Deletion Patient 1 [10]	Deletion Patient 2 [11]	Deletion Patient 3 [12]	Present Patient
Molecular features	Various	11p15 loss of methylation	upd(7)mat	Segmental upd(7q)mat	3.7 Mb chr7:127599298-131471494 del *denovo* paternal chromosome affected	2.8 Mb chr7:127889335-130708391 del *denovo* paternal chromosome affected	79 kb chr7:130071998-130151083 (*CEP41*, *MEST*, *MESTIT1*, *MIR335*, *COPG2*) Paternally inherited	*MEST*:c.890 + 1G > A
*MEST* (hg19: chr7:130131899-130146138) within deleted region			Yes	Yes	Yes	Yes	Yes	
Major clinical features								
Birth weight/height ≤ −2SD	91.7% (60)	100% (35)	72.7% (11)	2/4	−1.58 SD	−3.05 SD	−3.1 SD in length	−2.7 in weight, −3.5 in length
Postnatal height ≤ −2SD	84.2% (317)	83.80% (173)	80.9% (47)	4/4	−0.74 SD	4 years 9 months: −1.67 SD; 17 years: −1.23 SD	given < 2 SD from MPTH	6 months: SDS −4.5
Feeding difficulties	70.4% (307)	71.7% (173)	87.2% (47)	4/4	+	+ (first months only)	+	+
Relative macrocephaly (head circumference at birth at least 1.5 SD above birth weight and/or length)	85.7% (209)	99.1% (112)	85.2% (27)	4/4	−1.98 SD	4 years 9 months: −2.75 SD; 17 years: −3.13 SD	+ (when compared to length, not weight)	Not assessable (birth HC unavailable)
Skeletal asymmetry	57.3% (473)	77.40%	29% (62)	0/4	−	−	−	−
Craniofacial features								
Triangular face	93.9% (164)	98.7% (74)	50.0% (16)	3/3	Slightly	Slightly	+ (infancy and early childhood)	Slightly
Prominent forehead	88.1% (201)	93.7% (126)	100.0% (27)	NR	+	+	+ (infancy and early childhood)	+
Ear anomalies (low set)	49.3% (266)	50.0% (140)	68.8% (48)	3/4	+	Large ears with unfolded helix	−	−
Downturned corners of the mouth	47.7% (176)	57.0% (114)	25.7% (39)	2/4	−	−	+	−
Other features								
Fifth finger clinodactyly	74.6% (319)	80.7% (176)	56.3% (48)	2/4	−	−	−	− (Short fingers)
Muscular hypotonia	56.3% (103)	67.2% (61)	47.4% (19)	1/3	+ (severe truncal hypotonia)	−	−	−
Heart defects	NR	NR	NR	NR	Pulmonary stenosis	Interventricular septal defect	−	−
Scoliosis	17.6% (227)	10.0% (97)	16.3% (43)	NR	NR	−	+	−
Hearing impairment	NR	NR	NR	NR	mild hearing impairment	moderate hearing impairment requiring hearing aids	−	Transient hearing screening failure; resolved on follow-up.
Irregular spacing of teeth	36.9% (195)	28.6% (105)	38.9% (36)	0/3	−	−	+	−
Squeaky voice	45.2% (42)	39% (26)	71.0% (7)	1/4	−	−	−	−
Syndactyly	29.9% (264)	41.8% (141)	16.7% (48)	1/3	−	−	−	−
Café au lait naevi	NR	NR	NR	1/3	−	−	One café-au-lait spot on her hip	−
Development								
Motor/neuropsychological delay	36.6% (254)	30.5% (141)	58.3% (36)	1/3	+	+	−	−
Speech delay	39.7% (189)	31.7% (101)	63.9% (36)	NR	+ (severe)	+	−	−

Note: *n* denotes total no. of patients; + denotes present; − denotes absent; NR denotes not reported; MPTH denotes mid-parental target height.

## Data Availability

The original contributions presented in this study are included in the article. Further inquiries can be directed to the corresponding author.
